# Mortality Report for Month of December

**Published:** 1872-01-15

**Authors:** John H. Rauch

**Affiliations:** Sanitary Sup’t.


### MORTALITY.

MONTH OF DECEMBER, 1871.

| Year. | December. | Total. | S.-pox, Dec. | Total. | Pop'n. |
|---|---|---|---|---|---|
| 1862 | 203 | 2,578 | 3 | 5 | 137,000 |
| 1863 | 340 | 3,253 | 26 | 115 | 150,000 |
| 1864 | 263 | 4,044 | 12 | 283 | 161,000 |
| 1865 | 333 | 3,663 | 0 | 57 | 178,000 |
| 1866 | 309 | 5,931 | 2 | 9 | 200,418 |
| 1867 | 409 | 4,648 | 28 | 123 | 225,000 |
| 1868 | 375 | 5,984 | 2 | 147 | 252,054 |
| 1869 | 439 | 6,490 | 0 | 17 | 280,000 |
| 1870 | 415 | 7,342 | 0 | 15 | 299,000 |
| 1871 | 529 | 6,976 | 47 | 74 | 300,000 |

SMALL-POX.

The following table will show the number of cases reported for the months of November and December by wards; also the number of deaths for December:

CASES OF SMALL-POX REPORTED DURING THE MONTHS OF NOVEMBER AND DECEMBER, 1871.

| Wards. | Nov. | Dec. | | Deaths Dec. |
|---|---|---|---|---|
| 1 | — | 1 | | — |
| 2 | — | — | | — |
| 3 | 1 | 13 | | 2 |
| 4 | 2 | 7 | | 1 |
| 5 | — | 16 | | 2 |
| 6 | — | 13 | | 3 |
| 7 | 21 | 41 | | 14 |
| 8 | 23 | 45 | | 6 |
| 9 | 3 | 23 | | 1 |
| 10 | — | 5 | | — |
| 11 | — | 5 | | — |
| 12 | — | 5 | | — |
| 13 | 2 | 3 | | 1 |
| 14 | — | 2 | | — |
| 15 | 2 | 20 | | 5 |
| 16 | 3 | 7 | | 3 |
| 17 | 2 | 7 | | 1 |
| 18 | 4 | 2 | | — |
| 19 | — | 2 | | — |
| 20 | 1 | — | | — |
| Home of Friendless | 1 | — | S.-pox Hosp. | 8 |
| From Blue Island | 1 | — | | |
| Marine Hospital | 1 | — | Total | 47 |
| N. W. Depot | 1 | 2 | | |
| Barracks | — | 1 | | |
| Totals | 68 | 223 | | |

Deaths by Small-pox in Wards for Dec., 1871.

It will be seen from the above that small-pox has prevailed most in the 7th, 8th, 9th and 18th wards. In fact, nearly one-half of the cases occurred in the district south of Twelfth street on the West Side, the same being the case with regard to the mortality, particularly in the 7th ward, where the disease first existed, and where the fatality by it was much greater than in any portion of the city, the death-rate being one in three here, while it was one in five and a half for the remaining portion of the city. More deaths have occurred by this disease for this month than for the same time in the previous history of the city.

Of these there were 26 males and 21 females, 19 foreigners, 17 of foreign parentage born in Chicago, 12 born in the United States, 19 under five years, five from five to 20, 19 from 20 to 50, and four from 50 to 70. The first 19 had never been vaccinated, and of the remainder it is safe to say that if they had been re-vaccinated they would not have died. From this table it is to be seen that persons of all ages are affected by this disease, and that it is no respecter of ages.

The mortality for 1871 by small-pox has been 74, a less number than in any city of the same population in the United States. In Milwaukee for the month of December there were 65 deaths by small-pox and 184 cases reported, a mortality of one in two and five-sixths, while in the same month there were 225 cases reported here, with a mortality of 47, or one in about four and three-fourths. The total mortality for the year was 6,976: For January, 451, February, 400, March, 470, April, 421, May, 525, June, 558, July, 980, August, 818, September, 672, October, 440, November, 516 and December 525.

JOHN H. RAUCH,
Sanitary Sup't.

A method of applying dry heat and cold, suggested by Dr. Roberts, of Manchester, consists in the arrangement of a continuous coil of thin rubber tubing on a backing of canvas, to which it is to be cemented. One end of the tube can be connected with an elevated vessel containing water of the temperature desired, and the other end placed in a receiving vessel. The pad is to be applied to the surface the temperature of which it is desirable to modify.—*Br. Med. Jour.*

Primary Amputation of Thigh in a child One Year and a Half Old, with recovery and discharge from hospital in good health on the forty-first day, is recorded in *The Lancet* of Dec. 2d.

